# Book Review: Peacebuilding in Language Education: Innovations in Theory and Practice

**DOI:** 10.3389/fpsyg.2021.735738

**Published:** 2021-07-30

**Authors:** Yirong Xiong, Shan Chen

**Affiliations:** Department of International Education, Changde Vocational Technical College, Changde, China

**Keywords:** language education, book review, theory, practice, peacebuilding

“We live in a world today where peace is unprofitable.” (p. xxv) echoes the essence of peace in our life and makes the cornerstone of this compendium. The primary objectives of this innovative volume, entitled *Peacebuilding in Language Education: Innovations in Theory and Practice*, edited by Rebecca L. Oxford, María Matilde Olivero, Melinda Harrison and Tammy Gregersen, are to share peace theoretical frameworks and applications in English as a foreign language (EFL) or English as a second language (ESL), illuminate how to boost teaching, learning, and surviving by integrating peacebuilding into instruction and daily life, cater for contextualized information and transformative strategies that can be utilized to extend the multidimensional aspects of peace, and encourage readers to conduct research that encompass ideas and activities from this volume. Moreover, this volume aims to incorporate and actualize different dimensions of peace (inner, interpersonal, intergroup, international, intercultural, or ecological peace) in our daily life.

Following a Forward and an Introduction, this anthology includes 16 chapters, subsumed under five sections. Section I contains three chapters. Chapter 1 provides a panoramic view of the objectives of the volume, theoretical underpinnings, and outline of the book and calls for peace in EFL/ESL classrooms. Chapter 2 presents some foundational strategies for language teacher educators to become peacebuilders. In so doing, this chapter fleshes out the rudimentary peace knowledge and its four competencies (ethnocultural empathy, intercultural understanding, cognitive flexibility, and emotion regulation) which teachers need to master to enhance peace in their classes. Chapter 3 concentrates on the nonverbal and verbal aspects of peace communication in the classrooms. It highlights that harmony seekers embark not only on their words but also on their gestures, postures, facial expressions, etc. to nurture and nourish peace.

Section II, including four chapters, deals with inner, interpersonal, and intergroup peace. Delving into the role of inner peace and emotion regulation in the Argentinean context is the focus of Chapter 4. It specifically focuses on how language teacher educators prepare future teachers by helping them regulate their emotions and stay peaceful when they encounter the stress of using L2 orally. Chapter 5 highlights the role of different factors that lead to inner and interpersonal peace in the EFL/ESL classrooms, by endorsing Cooperative Open Learning (COOL), a holistic teaching approach. Chapter 6 probes into an underappreciated topic in applied linguistics; that is, how a revolutionary type of love can be related to peace in the process of teacher's professional identity development, by reporting on the trajectories of a Brazilian teacher of English. Chapter 7 brings to the fore that “difference” can be a source of strength that can contribute to unexpected unity through foregrounding transformative learning and peacebuilding activities. The authors conclude that “Peacebuilders offer compassionate words, maintain awareness of body language or advocate visibly and locally for equity and human rights. Peacebuilding fosters transformative learning and initiates internal changes in an individual” (p. 124).

Chapters 8, 9, and 10 comprise Section III, discussing the pivotal role of intercultural and international peace. Set in the US government institutes, Chapter 8 presents illuminating insights on how governmental institutions such as Foreign Service Institute (FSI) and the National Aeronautics and Space Administration (NASA) that do not concentrate on peace as an objective can teach languages for peace by employing defined language-learning models. The authors argue “language study has changed them in many positive ways, one of which is that it has predisposed them to peace” (p. 143). The authors in Chapter 9 accentuate that “Peace education research is at the crossroads of peace, education and Research” (p. 158), by unraveling how international faculty and international students can boost peace through enhancing their intercultural competence. Drawing on the tenets of critical language awareness and equity pedagogy, the last chapter in this section explores the role of peacebuilding through social justice pedagogies for Muslim immigrants by asking them to critically think about their identities and encouraging them to participate in discourse communities in ways that let learners co-construct or reconstruct narratives or counter-storytelling to support them as they resist Islamophobia.

Four chapters are subsumed under Section IV, which pertains to the implementation of peacebuilding through positive psychology (PP). This section innovatively integrates peace, positivity, and language. Chapter 11 argues that peace is a missing link in the PP, and like well-being, peace leads to the nurturing and nourishing of conditions that help individuals flourish. Besides, this chapter offers not only a refreshing and sound theoretical background but also realistic applications for our lives. Chapter 12 makes a demarcation between hate speech and hate crimes, both of which are pernicious. The author suggests that the significant factors to defy the challenges of hate can be experiential learning of empathy, education, and knowing individuals from the hated group. The authors in Chapter 13 present such negative words as “negative 3-H,” namely *hate, hurt* and *harm* to inform readers that we can go beyond negativity by utilizing some practical activities. Unlike the previous chapter, highlighting the negativity, the authors in Chapter 14 concentrate on “positive 3-H,” including *hope, help* and *harmony*, by providing classroom-friendly activities.

Section V includes two chapters. Chapter 15 is truly dedicated to peace-fostering activities to enhance inner, interpersonal, intergroup, intercultural, international, and ecological dimensions of peace. These engaging and thought-provoking activities include holistic instruction, experiential education, contemplative inquiry, and social awareness. The closing chapter brings together the principal themes, synthesizes the theoretical and practical contributions, and calls for more research to continue this line of inquiry.

This thought-provoking compendium, written by 23 scholars, teachers, and language practitioners, showcases numerous instructional, geographical, and cultural contexts. The volume, replete with user-friendly and practical classroom-based peacebuilding activities, reconciles theory and practice in a genuinely innovative manner. This anthology provides plenty of food for peace linguists, teachers, teacher educators, researchers, and educators with similar roles in other disciplines.

## Author Contributions

All authors listed have made a substantial, direct and intellectual contribution to the work, and approved it for publication.

## Conflict of Interest

The authors declare that the research was conducted in the absence of any commercial or financial relationships that could be construed as a potential conflict of interest.

## Publisher's Note

All claims expressed in this article are solely those of the authors and do not necessarily represent those of their affiliated organizations, or those of the publisher, the editors and the reviewers. Any product that may be evaluated in this article, or claim that may be made by its manufacturer, is not guaranteed or endorsed by the publisher.

